# Vestibular prognosis in idiopathic sudden sensorineural hearing loss with vestibular dysfunction treated with oral or intratympanic glucocorticoids: a protocol for randomized controlled trial

**DOI:** 10.1186/s13063-020-04579-6

**Published:** 2020-07-22

**Authors:** Weiming Hao, Liping Zhao, Huiqian Yu, Huawei Li

**Affiliations:** 1 ENT Institute and Otorhinolaryngology Department of Eye & ENT Hospital, State Key Laboratory of Medical Neurobiology and MOE Frontiers Center for Brain Science, Fudan University, 83 Fenyang Road, Shanghai, 200031 People’s Republic of China; 2 NHC Key Laboratory of Hearing Medicine (Fudan University), Shanghai, 200031 People’s Republic of China; 3 Institutes of Biomedical Sciences, Fudan University, Shanghai, 200032 People’s Republic of China; 4 The Institutes of Brain Science and the Collaborative Innovation Center for Brain Science, Fudan University, Shanghai, 200032 China

**Keywords:** Randomized controlled trial, Vestibular function, Sudden hearing loss, Glucocorticoid

## Abstract

**Background:**

Idiopathic sudden sensorineural hearing loss (ISSNHL) is a rapid-onset sensorineural hearing impairment with unclear etiology and unsatisfying treatment effects. Vestibular dysfunction has been considered as a poor indicator in the clinical manifestations and prognosis of ISSNHL, which occurred in approximately 28–57% cases. Glucocorticoids, administered through oral or intratympanic way, are currently regularly and standardly applied for ISSNHL to improve the hearing outcome. However, the vestibular prognosis of ISSNHL after routine treatments remains seldom explored. This study aims to compare the effectiveness of oral and intratympanic glucocorticoids in ISSNHL with vestibular dysfunction in terms of the pattern and trajectory of possible process of vestibular function recovery.

**Methods/design:**

A randomized, outcome-assessor- and analyst-blinded, controlled, clinical trial (RCT) will be carried out. Seventy-two patients with ISSNHL complaining of vestibular dysfunction appearing as vertigo or imbalance will be recruited and randomized into either oral or intratympanic glucocorticoid therapy group with a 1:1 allocation ratio. The primary outcomes will be vestibular function outcomes assessed by sensory organization test, caloric test, video head impulse test, cervical vestibular evoked myogenic potential, and ocular vestibular evoked myogenic potential; the secondary outcomes include self-reported vestibular dysfunction symptoms; dizziness-related handicap, visual analogue scale for vertigo and tinnitus; and pure tone audiometry. Assessments of primary outcomes will be performed at baseline and at 4 and 8 weeks post-randomization, while assessments of secondary outcomes will be performed at baseline and 1, 2, 4, and 8 weeks post-randomization.

**Discussion:**

Previous intervention studies of ISSNHL included only hearing outcomes, with little attention paid on the prognosis of vestibular dysfunction. This trial will be the first RCT study focusing on the progress and prognosis of vestibular dysfunction in ISSNHL. The efficacy of two commonly used therapies of glucocorticoids will be compared in both auditory and vestibular function fields, rather than in the hearing outcome alone.

**Trial registration:**

ClinicalTrials.gov NCT03974867. Registered on 23 July 2019

## Background

Sudden sensorineural hearing loss (SSNHL) is a rapid-onset inner ear disease. It is defined as a sensorineural hearing loss of at least 30 dB over at least three test frequencies occurring within 72 h [1, 2]. The reported incidence rate of SSNHL is about 5–27/100,000 people per year [1], which has been widely considered underestimated because of the unregistered cases with out-of-hospital spontaneous recoveries. The etiology in about 71 to 90% of SSNHL cases remains uncertain, which is defined as idiopathic SSNHL (ISSNHL) [1, 3]. Various postulated etiological theories have been proposed including microvascular impairment, viral infections, inner ear electrolytic disorders, trauma, autoimmune diseases, and central nervous system (CNS) diseases [3–7]. Based on the close anatomic relationship between cochlea and vestibule, approximately 28–57% of ISSNHL patients have also reported of co-occurring symptoms of vertigo [8].

There are two administration patterns of glucocorticoids for ISSNHL: systemic (oral or intravenous) and intratympanic therapies. Compared with the traditional oral administration, intratympanic therapy is thought to be superior for its (1) bypassing the blood-labyrinth barrier and achieving higher drug concentrates in the inner ear and (2) avoiding most of the systemic side-effects of glucocorticoids. A well-designed randomized trial was conducted in 2011 by Rauch and his colleagues, which supported the non-inferiority in hearing outcomes of intratympanic therapy compared with oral prednisone in ISSNHL. However, the study was failed to reject the inferiority based on the 10-dB non-inferiority standards in the subgroup analysis of ISSNHL with dizziness [9].

Considering that few investigations have been carried out on progress and prognosis of ISSNHL-related vestibular dysfunctions after basic treatment, we designed this randomized, assessor- and analyst-blinded, controlled trial with two interventional arms, one is the oral glucocorticoid therapy group and the other one is the intratympanic glucocorticoid therapy group. Our research hypothesis is that intratympanic methylprednisolone is superior to oral prednisone on vestibular function recovery of ISSNHL patients with vertigo.

## Methods/design

### Study design and settings

This protocol is reported in accordance with the *Standard Protocol Items: Recommendations for Interventional Trials guidelines* (*SPIRIT*) (Additional file 1) [10]. This study is designed as an 8-week, single-center, randomized, assessor- and analyst-blinded, controlled trial with two parallel interventional groups in a 1:1 allocation. Patients will be recruited from outpatient clinics of the Eye and ENT Hospital of Fudan University in Shanghai, qualified with well-trained doctors, staff, and required facilities for this clinical trial.

### Participants

Patients who meet all of the following inclusion criteria will be considered eligible:

1. Adults aged between 18 and 70 years old;
2. Diagnosed with unilateral ISSNHL according to the National Institute for Deafness and Communication Disorders (NICDC) criteria [2];
3. Complained of vertigo/imbalance together with abnormal results in at least one of the vestibular function tests, including sensory organization test (SOT), caloric test, video head impulse test (vHIT), cervical vestibular evoked myogenic potentials (cVEMP), and ocular vestibular evoked myogenic potentials (oVEMP);
4. Onset of audio-vestibular symptoms occurred within 7 days; and
5. Be willing to sign the informed consent of the study.

Patients with any of the following conditions will be excluded:

1. Definite etiologies are found or highly suspected after clinical evaluations, such as vestibular schwannoma, stroke, trauma, or demyelinating disease;
2. Diagnosed with a present or previous hearing or balance disorders, such as Meniere’s disease, benign paroxysmal positional vertigo, vestibular neuronitis, vestibular migraine, otosclerosis, luetic, and congenital or genetic hearing loss;
3. Pure tone audiometry (PTA) threshold of the unaffected ear is higher than 25 dB;
4. Present with conditions contraindicated systemic glucocorticoid use, such as tuberculosis, hepatitis B or C infection, active herpes zoster infection, pancreatitis, insulin-dependent diabetes mellitus, severe osteoporosis, or gastrointestinal ulcer;
5. A history of more than 3 days sufficient systemic glucocorticoid uses (≥ 1 mg/kg/day) within 3 months;
6. Not appropriate for receiving vestibular function tests;
7. Multiple organ dysfunction or unstable vital signs;
8. Pregnancy or lactation; and
9. Unsuitable for the trial because of any other reasons identified by investigators.

### Recruitments and randomization

Patients who visit doctors from the outpatients and suspected of ISSNHL with vestibular dysfunction will be screened for eligibility. Eligible patients who consent to participate will receive either oral prednisone or intratympanic methylprednisolone randomly in a 1:1 allocation. The randomization sequence will be generated through @RANDOM.ORG, an internet website (https://www.random.org) producing true random sequence according to atmosphere noise. The concealed randomization sequence file will be constructed and kept in sequentially numbered, sealed, opaque envelopes by a staff member (Zhao L.) in the Eye and ENT Hospital of Fudan University outside the study team. All the potential participants will be evaluated in strict chronological order of visiting time. When a patient is officially enrolled and numbered, the staff member will be contacted by telephone and open the corresponding envelope to find the randomized treatment for this patient. Assessors in examination rooms and statistician analysts are not allowed to receive any information of the group allocation. The information of participants will be referred using research code among investigators, and their personal information (like name and contacts) will be kept confidential before, during, and after the trial.

Following randomization, baseline information of the participants in demographic and clinical characteristics will be collected, such as age, gender, nationality, occupation, date of onset, predisposition, initial symptoms, time of diagnosis, any treatments before enrolments, body mass index (BMI), and comorbidities. The objective vestibular function evaluated by SOT, videonystagmography (VNG), vHIT, cVEMP, and oVEMP and the hearing outcome assessed by PTA will be performed at baseline and at 4 and 8 weeks after randomization, while the subjective vestibular dysfunction feelings reflexed by dizziness handicap inventory (DHI) and visual analogue scale for vertigo (VAS-V) and the tinnitus condition assessed by VAS in tinnitus (VAS-T, visual analogue scale for tinnitus) will be performed at baseline and at 1, 2, 4, and 8 weeks after randomization. Caloric test is routinely included in VNG. Acoustic impedance and magnetic resonance imaging of the internal auditory canal (MRI-IAC) will be performed only once at the baseline examination to exclude the potential misdiagnosed SSNHL.

### Interventions and comparisons

Once a participant is randomized, the treatment procedure starts immediately (Table 1). The participants in group 1 will receive oral prednisone 1 mg/kg/day (maximum daily dosage is no more than 60 mg) for 7 days, followed by a 7-day taper (Table 1). The patients are advised to take the medicine in 30 min before every breakfast, and not to divide the doses. The participants in group 2 will receive 7 intratympanic 40 mg/ml methylprednisolone injections in 14 days, one injection every other day.

**Table 1:** Glucocorticoid therapy protocol in each group

	Drug	Protocol
Group 1	Pred.	Glucocorticoid therapy: d1–d7: Oral Pred. 1 mg/kg/d (maximum daily dosage is no more than 60 mg) d8–d9: Oral Pred. 10 mg less than d7 d10–d11: Oral Pred. 10 mg less than d9 d12: Oral Pred. 10 mg less than d11 d13: Oral Pred. 10 mg less than d12 d14: Oral Pred. 10 mg less than d13^a^
Group 2	Met.	Glucocorticoid therapy: One intratympanic injection of 40 mg/ml Met. at d1, d3, d5, d7, d9, d11, and d13;^b^

Pred. prednisone, Met. methylprednisolone, d. day

^a^If the patient’s weight is less than 50 kg, the administration will be stopped after the day with < 10 mg prednisone administered, for example, a patient weighs 45 kg will stop receiving glucocorticoids at the 13th day

^b^One day early or late of injection is allowed for practicality

The otolaryngologists in charge of the injection work are asked to inject at the posterior superior quadrant and fulfill the tympanic cavity using operating microscopes, after lidocaine spray anesthesia. Patients will be asked to keep supine position with the affected ear slightly up to 30° and avoid swallow during injection and in 30 min after the injection. The drug for injection is methylprednisolone sodium succinate for injection by Pfizer Manufacturing Belgium NV.

Of all the participants, the length of treatment would be extended for another week if the change in hearing threshold (average dB in PTA) is less than 10 dB, or average of PTA is worse than 55 dB in the patient’s affected ear, under the participant’s agreement. The salvage treatment regimen is 3 injections of 40 mg/ml methylprednisolone every other day. Records and effects of the salvage treatments will be reported in the study results and evaluated according to both intention-to-treat (ITT) and per-protocol analyses. For those with severe vertigo attacks, drugs for symptom control like mannitol or diazepam can be used temporarily.

### Outcome measurements and follow-ups

In Table 2, criteria are listed in detail to distinguish the central vestibular system (CVS) compensation and peripheral vestibular system (PVS) restoration according to the following evidence:

1. SOT is a measurement for dynamic and static posturography, which may figure out the different roles of the somatosensory, visual, and vestibular systems. The vestibular scores in SOT will help us to distinguish peripheral function self-restoration from CVS compensation.
2. The caloric test has been used to evaluate the lateral semicircular canal function with a good sensitivity [11]. In the caloric test, unilateral weakness (UW), directional preponderance (DP), and spontaneous nystagmus (SpN) are three parameters to judge the different phases of central vestibular compensation. UW presents at all three phases of acute injury, static compensation, and dynamic compensation phase and DP presents at the acute injury phase and static compensation phase, while SpN only presents at the acute injury phase [12]. Meanwhile, the caloric test results of complete function restoration in PVS should be the same as those in normal individuals: absence of UW, DP, or SpN.
3. vHIT can be employed for evaluating horizontal and vertical semicircular canals and has been taken as a specific indicator of peripheral vestibular function [13, 14]. The decreased gains and corrective saccades are signs of the corresponding semicircular canal dysfunction.
4. cVEMP is a widely used measurement for assessing the saccular and inferior vestibular pathway functions, while oVEMP for evaluating the utricular and superior vestibular pathway functions [15]. Based on the integrity of neural pathways, we may reasonably assume that if the dysfunction of otolith organ and its afferent pathway has not recovered in our participants, the compensation effects of CVS will not bring a normal VEMP result [14, 16].

**Table 2:** Difference in vestibular function tests between compensation of the central vestibular system and restoration of the peripheral vestibular system

	Compensation of CVS	Restoration of PVS
Subjective complaints	Normal	Normal
SOT	Abnormal	Normal
Caloric test	Static compensation: UW+ DP+ Dynamic compensation: UW+ DP−	UW− DP−
vHIT	Abnormal	Normal
cVEMP/oVEMP	Abnormal	Normal

*CVS* central vestibular system, *PVS* peripheral vestibular system, *UW* unilateral weakness, *DP* directional preponderance, *SpN* spontaneous nystagmus

Here come two possible patterns in vestibular dysfunction recovery of ISSNHL. The first pattern (pattern A) is that the patients undergo well central vestibular compensations with no recovery of PVS function. In this pattern, patients may show a quite normal ability of balance evaluated by subjective complaints, DHI or VAS-V; however, since the peripheral vestibular organs remain dysfunctional, vestibular test battery (i.e., SOT, caloric tests, vHIT, and VEMPs) will come out with abnormal results. The second possible pattern (pattern B) is that the PVS injuries are completely or partially restored in the patients. In this condition, not only the subjective complaints will disappear, but also the objective vestibular test results will get back to normal, or at least less abnormal.

#### Measurements

The methodology details of the following tests have been reported before [17].

##### SOT

The SOT in our study is performed with the Synapsys Posturography System (SPS, SYNAPSYS, Inc. Marseille, France). Six sensory test modes are performed with changing supports and visual conditions. The results are analyzed comprehensively to give a score for each sensory system (somatosensory, visual, and vestibular systems) and a composite score. Results are considered abnormal when the score is lower than the age-specific normative data.

##### Caloric test

We deliver the caloric test using the air caloric irrigator system of ICS AirCal (GN Otometrics, Taastrup, Denmark) and record eye movements using videonystagmography (VNG) of SYNAPSYS VNG Ulmer (SYNAPSYS, Inc. Marseille, France). Patients are placed in a supine position in a darkroom, with the head flexed at 30°. The temperature of the warm and cool air is 50 °C and 22 °C, respectively. The unilateral weakness (UW) and directional preponderance (DP) are used to quantify the difference between the caloric responses of the two ears. The abnormal caloric result is defined as an absolute value of UW% greater than 22% and/or an absolute value of DP greater than 27%.

##### vHIT

ICS Impulse system (GN Otometrics, Taastrup, Denmark) will be used for vHIT in this study. The examiner performs head impulses (150 to 200°/s peak head velocity) randomly in unpredictable timing and direction in the plane of each canal (the horizontal, anterior, and posterior semicircular canal), and at least 15 impulses for each side are acquired. The software analysis algorithm calculates the vestibular-ocular gain. Normal gain is defined as > 0.8 for the lateral canals and > 0.7 for the vertical canals. The pathological saccade is defined as refixation saccades categorized as either covert saccades (occurring during a head movement) or overt saccades (occurring after a head movement) [18]. The result of a semicircular canal will be considered abnormal when there are pathological saccades and the gain is out of normal range.

##### VEMP

The recording device of both cVEMP and oVEMP is the Bio-logic Navigator PRO (Natus Medical Inc., San Carlos, USA).

##### cVEMP

Patients are asked to lie in supine position on a bed, with head raised to 30 to 45° away from the bed during recording to ensure good muscle tone. Electrodes are placed as operation manual. Air-conducted sound with 500-Hz short tone bursts (2 ms rise/fall time and 2 ms plateau time) are presented through insert headphones as stimuli. The starting stimulus intensity is 95 dB nHL and decreases by 5 dB nHL each time until the meaningful wave is undetectable. The lowest intensity with a characteristic waveform is defined as threshold. The result will be considered abnormal if one of the following conditions is met: (1) the amplitude asymmetry ratio (AR) is more than 37%; (2) the cVEMP meaningful waveform (where the waveforms with positive-negative-positive peak, P1-N1-P2, could be recognized and well repeatable) is absent at 95-dB nHL stimulus or the threshold is out of range compared with age-specific normative data; (3) delayed response: the cVEMP threshold shift is out of the range: P1 range 15.66 ± 7.22; N1 range 23.42 ± 5.18 (the mean of normal range ± 2 × SD) [19–22].

##### oVEMP

The recording device, software, and stimuli are the same as those in cVEMP. The patients remain lying supine and are asked to look upward (approximately 30° above the horizontal plane) during recording. Electrodes are placed as reported in previous studies [17]. The recording procedure is the same as that in cVEMP, and the lowest intensity with a characteristic waveform is defined as threshold. We define the abnormal result of oVEMP as (1) absence of meaningful waveform (where the waveforms with negative-positive peak, N1-P1, could be recognized and well repeatable); (2) the threshold out of range compared with age-specific normative data; and (3) amplitude asymmetry ratio (AR) ≥ 40% [19, 20].

All examinations will be performed by trained physicians skilled at neuro-otological tests.

##### DHI and VAS

The dizziness handicap inventory (DHI) is a self-assessment questionnaire with 25 items, and the reliability of the Chinese version has been verified [23–26]. The 25 items can be divided into 3 subscales: physical, emotional, and functional aspects, and the total scores range from 0 to 100. The higher the score, the more severe the dizziness is in the patients.

Visual analogue scale is a universal psychometric scale evaluating subjective attitudes [27, 28]. When applied in vertigo or tinnitus (VAS-V or VAS-T), respondents specify their level of vertigoes or tinnitus by indicating a position along a continuous line between two endpoints without marked scale. A score from 0 to 10 will be made based on the length of the line. The higher the score, the more severe the symptom is.

#### Primary outcomes

To evaluate the recovery of vestibular function, we set the recovery rates of the whole battery of vestibular function tests (SOT/caloric test/vHIT/VEMPs) as the primary outcome, which is the proportion of patients whose abnormal results of vestibular function tests at baseline recover to normal at 4-/8-week follow-up:

$$ \mathrm{recover}\mathrm{y}\ \mathrm{rate}=\frac{\begin{array}{c}\mathrm{number}\ \mathrm{of}\ \mathrm{patients}\ \mathrm{recover}\ \mathrm{from}\ \mathrm{abnormal}\ \mathrm{result}\ \mathrm{at}\ \mathrm{baseline}\\ {}\ \mathrm{to}\ \mathrm{normal}\ \mathrm{at}\ 4-/8-\mathrm{week}\ \mathrm{follow}-\mathrm{up}\end{array}}{\mathrm{number}\ \mathrm{of}\ \mathrm{all}\ \mathrm{enrolment}\ \mathrm{participants}}\times 100\%. $$

#### Secondary outcomes

Secondary outcomes include the change of subjective evaluations in vestibular, tinnitus, and hearing assessments:

1. Change of DHI and VAS-V, VAS-T scores: change of DHI and VAS-V scores from baseline at 1-, 2-, 4-, and 8-week follow-up.
2. Change of PTA: change of average of PTA from baseline at 1-, 2-, 4-, and 8-week follow-up; in this study, we define a 10-dB PTA criterion as clinically significant difference based on a previous RCT [9].
3. Safety: rates of study-related adverse events (AEs), such as short-term systemic use of glucocorticoids related SAEs and AEs, and intratympanic injection-related AEs.

### Time points

The study included 5 follow-up visits in total: a baseline visit to sign the consent and to record the baseline information, 2 visits during treatment interval to record subjective vestibular questionnaires (DHI, VAS-V, and VAS-T) and hearing outcomes (PTA) and to monitor treatment safety outcomes, and 2 follow-up visits at 4 weeks and 8 weeks to assess hearing and vestibular functions and monitor safety outcomes. To optimize examination resources and reduce burden of examiners, among all five vestibular function tests (SOT, caloric test, vHIT, cVEMP, and oVEMP), only those with previous abnormal results will be repeated at follow-up visits.

**Fig. 1:**
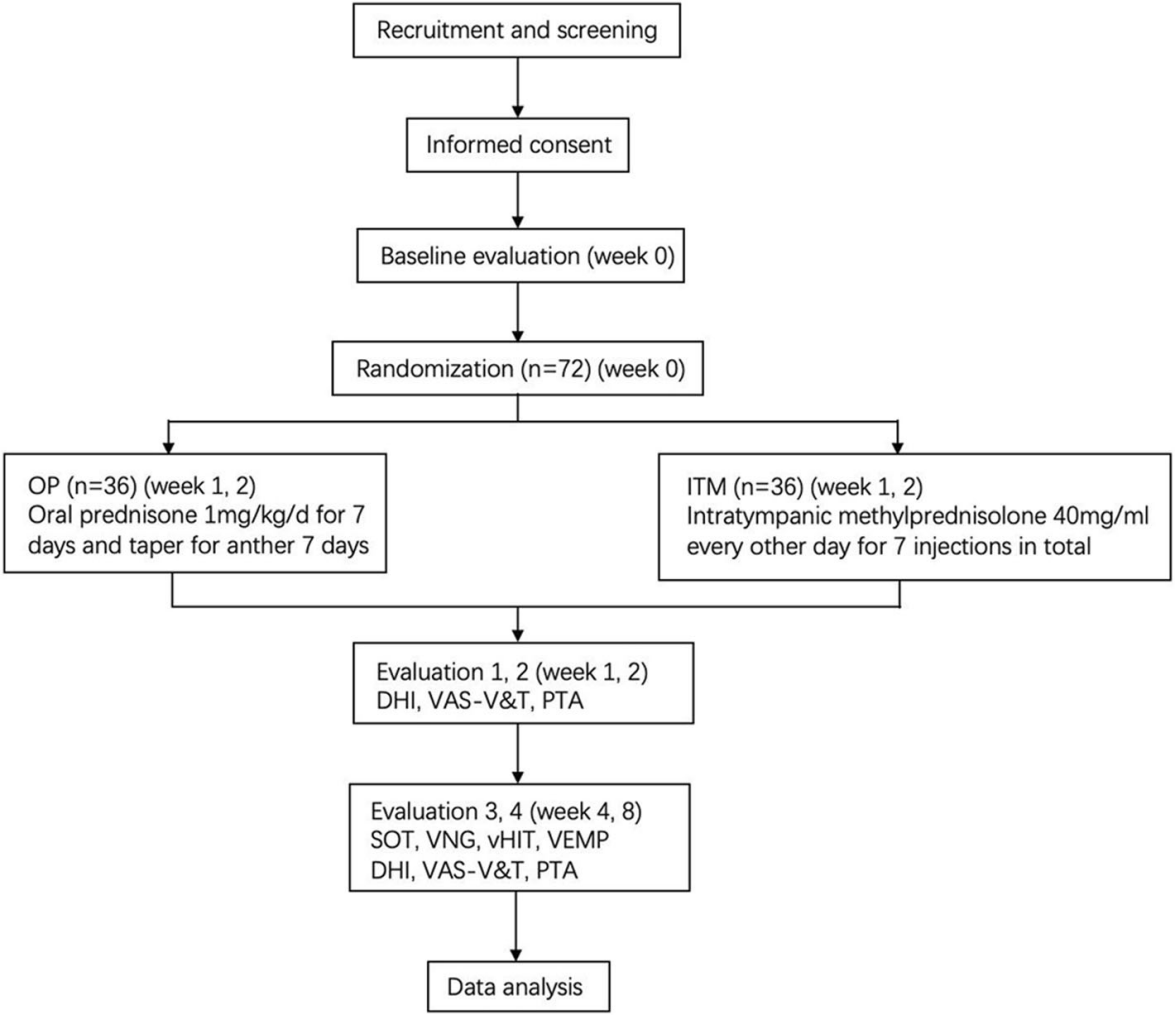
Study flow diagram (OP oral prednisone, ITM intratympanic methylprednisolone, DHI dizziness handicap inventory, VAS-V&T visual analogue scale for vertigo and tinnitus, PTA pure tone audiometry, SOT sensory organization test, VNG videonystagmography, vHIT video head impulse test, VEMP vestibular evoked myogenic potentials)

The study flow diagram is shown in Fig. 1, and the SPIRIT figure of enrolment, interventions, and assessments is presented in Table 3.

**Table 3:**
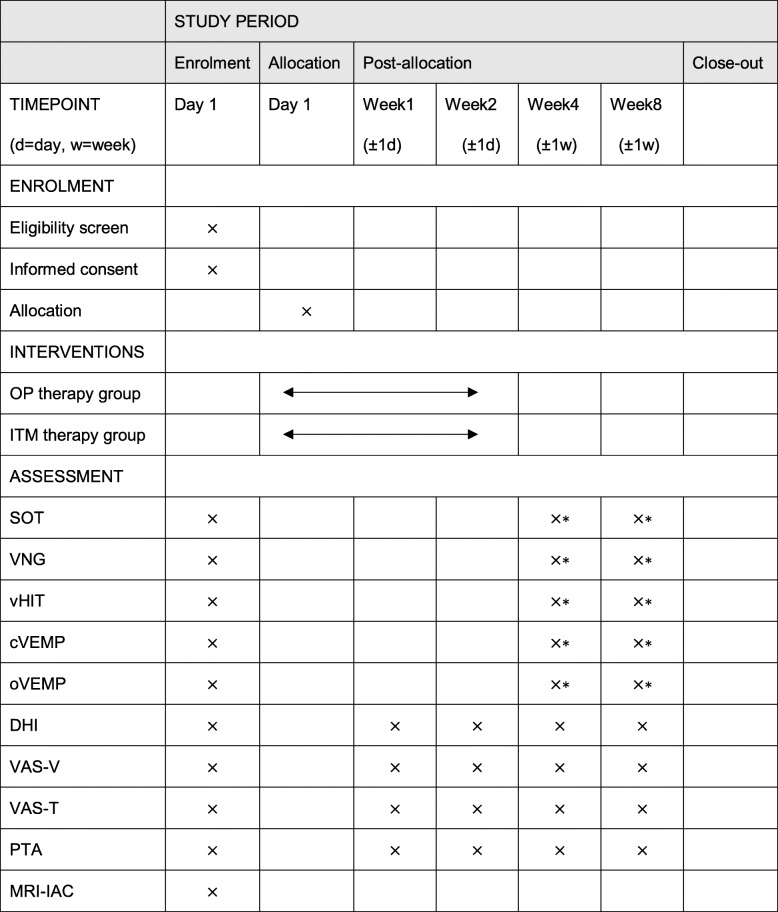
The SPIRIT figure of enrolment, interventions, and assessments

*VNG* videonystagmography which includes the caloric test, *MRI-IAC* magnetic resonance of the internal auditory canal

*A follow-up test is only repeated when the previous test results in abnormal findings

### Adverse events

Any untoward medical occurrences with unfavorable symptoms in the participants are defined as adverse events (AEs). AE is not necessarily to have a causal relationship with the treatment. If the AEs are life-threatening, result in death, persistent, and significant disability or incapacity, or make the participants’ hospitalization, we define them as serious adverse events (SAEs). The investigators will routinely ask the patients if there have been any unexpected symptoms. Study-related and non-study-related AEs will be recorded in the patient’s clinical history by doctors in clinics and then reported to a trial investigator (Yu H.). Participants experiencing AEs will be followed up until the end of the events or the end of the trial. Any SAEs during this trial will be reported to the trial steering committee (TSC) and Adverse Drug Reaction Administration (ADRA) of the Eye & ENT Hospital of Fudan University within 48 h at learning of the event.

We list some possible study-related AEs here:

1. Short-term systemic use of glucocorticoids related SAEs: fracture, sepsis, and venous thromboembolism [29];
2. Short-term systemic use of glucocorticoids related AEs: blood glucose problem, appetite change, sleep change, weight change; and
3. Intratympanic injection-related AEs: ear pain, ear infection, tympanic membrane perforation, and worse vertigo after injection.

### Participant withdrawal

The participants will be informed of the right to withdraw from the study at any time after consent. The enrolled participants who are found ineligible later, lost to follow-up, or withdraw consent for any reason will be regarded as withdrawal. As for those who decided not to receive the protocol intervention, we will ask them if they would like to continue with the follow-up visits and if they agreed, their results will be regarded as variation other than withdrawals in ITT analysis.

We provide an online Wechat and telephone contacts to all the participants for timely communication and health education, which will promote the participants’ retention and adherence to the intervention protocols. Furthermore, we optimized the follow-up process by setting a specialty clinic for SSNHL, minimizing the waiting time, and waiving extra examination and treatment fees.

### Blinding

We decide not to set blindness of the interventions to patients and doctors, because that setting blindness to patients and doctors will need placebo injections to some of the participants, which may bring unnecessary pain and tympanic membrane perforation risk to the patients. The specialists in test rooms and statistical analysts will be kept blinded during the trial until the statistical analyses are done.

### Trial management and quality control

A data monitoring committee (DMC) has been established to store, monitor, and check the authenticity, security, and integrity of the database. All members in the DMC are independent of the study sponsors and declared no competing interests. The DMC will periodically review the accumulated data and communicate the problems of its deliberations to the study team if necessary. The frequency of the interim analyses will be judged by the Chair of the DMC, based on the consultation with the TMC. We anticipate that there might be 2–3 interim analyses before the final analysis.

### Sample size and statistical methods

In our previous studies, we reported the poor indicator of the vestibular system lesion patterns in SSNHL and put forward that the incidence of vestibular dysfunction [30, 31]. However, we only found the existence of vestibular recovery in clinical case reports [16]. Due to lack of previous reported data on changes of vestibular function test results, this study sample size was calculated based on data from preliminary clinical practice in our hospital. Among patients who were diagnosed as ISSNHL and treated with oral prednisone, the recovery proportion of the vestibular function tests (measured by SOT, caloric test, or VEMPs) was 0.25, while the recovery proportion among those treated with intratympanic glucocorticoids was approximate 0.64. Using the program of G*Power 3.1 for Fisher’s exact test, we calculated a sample size of 30 per arm with 80% power at a two-tailed 5% level of significance [32]. To allow for 20% dropout, 36 participants per arm will be needed.

For the outcomes, categoric variables will be expressed as rates, while numerical variables as the mean ± SD. Baseline data will be compared between two groups with a chi-squared test for dichotomous samples and Student’s *t* test or Mann-Whitney *U* test for two independent samples where appropriate. For comparisons of recovery in 4 and 8 weeks from baseline, the recovery rates of vestibular function tests will be calculated by chi-squared tests, and logistic regression adjusts for potential confounders like age, initial PTA, the number of involved vestibular organs, MRI-IAC results, and other characteristics, while the numerical variables like UW of the caloric test, PTA, and scores in DHI, VAS-V, and VAS-T will be calculated by mixed-model with repeated measures analyses of variance (ANOVA), with group and time as fixed effects and subject as a random effect, controlling for the potential confounders. Between two intervention groups, the chi-squared test will be used to analyze the difference for dichotomous outcomes (e.g., recovery rates) and Student’s *t* test for two independent samples or Mann-Whitney *U* test where appropriate. A value of *p* < 0.05 of two-sided is considered statistically significant. We will calculate relative risk (RR) with corresponding 95% confidence intervals to compare dichotomous variables, and mean difference (MD) for continuous variables.

An ITT analysis and a per-protocol analysis will be both performed at each outcome. For the missing data in ITT analyses, if there is any, multiple imputation methods will be used. Up-to-date versions of SPSS (SPSS, Chicago, IL, USA) will be used to conduct analyses. A statistician from the Medical College of Fudan University will perform the analyses. Not until the statistician complete the analysis, will he be unblinded to the group allocations and the study hypotheses. All data sent to the analyst will be anonymized, and the study groups will be coded as groups A and B.

### Ethics and disseminations

This protocol and the template informed consent forms have been reviewed and approved by the institutional review board (IRB) and the Ethical Committee of Eye & ENT Hospital of Fudan University (Reference Number: 2017047). The TMC will make safety and progress reports to the IRB at least annually and within 3 months of study completion. Patients and the public were not involved in the design of this study. However, the study was initially discussed with ISSNHL patients’ representative on how best to involve patients throughout the proposed project. Finally, the study results will be informed to the public via peer-reviewed journals or academic conferences.

## Discussion

Vestibular dysfunction, commonly complained by patients as vertigo, dizziness, or lateropulsion, has been considered as a risk factor of profound hearing loss and poor prognosis [30–34]. Recently, researchers made their efforts to specify the lesion patterns of the vestibular system in ISSNHL and proposed that the utricle and superior vestibular pathway is the most vulnerable vestibular site in ISSNHL, followed by the lateral semicircular canal and superior vestibular pathway [30]. Severe vertigo and static imbalance markedly improve over a couple of days or weeks in most of patients, while some may suffer from long-term residual dizziness. Recovery of peripheral vestibular function and central vestibular compensation might be involved in this process. Vestibular function tests, such as SOT, caloric test, vHIT, and VEMP, are effective and objective methods to distinguish restoration of peripheral vestibular function and compensation of the central vestibular. It has been reported that in a few cases of SSNHL, function of otolith organs reflexed by VEMPs was absent at onset and recovered or improved after weeks to months [16]. These cases suggested the role of peripheral vestibular function restorations. When peripheral vestibular injury is failed to recover by itself, the central compensation will take place. However, due to few previous researches in this field, the roles and proportions of central compensation versus peripheral recovery remain uncertain.

We assume that appropriate treatments for ISSNHL may have favorable effects promoting the vestibular recovery process, as those in hearing outcomes. Glucocorticoid therapy is currently a regular and standard treatment for ISSNHL according to guidelines [1, 35]. The plausible mechanisms include anti-inflammatory, immune-suppressive, and inner-ear-homeostasis-related gene regulative effects [36]. Regrettably, with numerous studies of different evidence levels delivered in the past six decades, the effectiveness of glucocorticoids in treating ISSNHL remains uncertain. Most of the RCTs and meta-analyses on this topic claimed of disappointing results with little measurable improvements in glucocorticoids over placebo arms [37–39]. It is worth noting that many confounding factors like various regimens and administration of drugs, different time points of starting treatments, and different probable causes existed in these studies. Few studies have focused on the effects of glucocorticoids in vestibular recovery of ISSNHL. Taking the previous researches in acute unilateral vestibulopathy for reference, improvements have been found evaluated by vestibular function tests, the symptom loads, and DHI scores [40, 41]. Hereby, we may speculate that glucocorticoids could possibly accelerate vestibular function recovery via restoration of the peripheral vestibular system.

To assess the vestibular functions, we intend to perform a battery of vestibular function tests including (1) the SOT for assessing static and dynamic posture control ability of somatosensory, visual, and vestibular systems distinguishingly and comprehensively; (2) the caloric test for evaluating horizontal semicircular canal functions and superior vestibular integrity; (3) vHIT for evaluating functions of horizontal and vertical semicircular canals; and (4) cVEMP for investigating the saccular function and the inferior vestibular pathway and oVEMP for assessing utricular function and the superior vestibular pathway [11, 13, 15, 42, 43]. Moreover, we plan to perform DHI and VAS-V, two qualified and widely used subjective measurements, to assess the severity of symptoms and negative influence on patients’ daily lives [25, 44].

In conclusion, our study will be the first to assess and follow the vestibular function conditions of ISSNHL up using a whole battery of vestibular function tests. Based on our clinical practice experience, we hypothesize that the effects of intratympanic glucocorticoids will be superior to those of oral therapy in terms of the outcome measurements mentioned above. Moreover, we expect that participants in the intratympanic group will experience more significantly reduced DHI scores, lessened VAS-V scores, and better enhanced recovery of PTA.

## Trial status

The planned date of the first enrolment is 1 Sep 2019. The estimated time required for recruitment is 12 months. The total duration of this study is expected to be 18 months, including statistical analysis and article writing. This protocol version number is Ver.3.

## Supplementary information

**Additional file 1.** SPIRIT checklist.

**Additional file 2.** Case Report Forms Template.

**Additional file 3.** Adverse Event Forms Template.

## Data Availability

During the study, the datasets used in the current study are available from the corresponding author on reasonable request. After the study, the results of this trial will be published in peer-reviewed journals and presented at national and/or international conferences.

## Abbreviations

ISSNHL: Idiopathic sudden sensorineural hearing loss
RCT: Randomized controlled trial
CNS: Central nervous system
PNS: Peripheral nervous system
SPIRIT: Standard Protocol Items: Recommendations for Interventional Trials guidelines
ENT: Ear, nose, and throat
VEMP: Vestibular evoked myogenic potentials
cVEMP: Cervical vestibular evoked myogenic potentials
oVEMP: Ocular vestibular evoked myogenic potentials
SOT: Sensory organization test
vHIT: Video head impulse test
PTA: Pure tone audiometry
VNG: Videonystagmography
DHI: Dizziness handicap inventory
VAS: Visual analogue scale
VAS-V: Visual analogue scale for vertigo
VAS-T: Visual analogue scale for tinnitus
MRI-IAC: Magnetic resonance imaging of the internal auditory canal
AE: Adverse event
SAE: Severe adverse event
ITT: Intention to treat
UW: Unilateral weakness
DP: Directional preponderance
SpN: Spontaneous nystagmus
AR: Asymmetry ratio
DMC: Data monitoring committee
CRF: Case report form
RR: Relative risk
IRB: Institutional review board
