# Supplementary material for: Vestibular prognosis in idiopathic sudden sensorineural hearing loss with vestibular dysfunction treated with oral or intratympanic glucocorticoids: a protocol for randomized controlled trial
Source: Trials. 2020 Jul 22;21:669. doi: 10.1186/s13063-020-04579-6 (PMC7477872; doi:10.1186/s13063-020-04579-6)
Supplement: Supplementary file 2 — Additional file 2. Case Report Forms Template. [file 13063_2020_4579_MOESM2_ESM.pdf]

口服和鼓室内注射激素治疗特发性突聋伴眩晕患者前庭功能转归随机对照研究

Vestibular prognosis assessment of the idiopathic sudden sensorineural hearing loss with vestibular dysfunction treated with oral or intratympanic glucocorticoids: a randomized controlled trial

病例报告表

Case Report Form

中心编号：

患者编号：

1. 筛查 Screening

1.1. 知情同意书签署 Informed consent ☐

签署日期 Date of signing the informed consent：_______年____月____日(yyyy-mm-dd)

1.2. 纳入标准 Inclusion criteria

以下各条均需满足 Patients who meet *all* of the following criteria are eligible for enrollment:

**纳入标准 Inclusion criteria**	**是 Yes**	**否 No**
1.年龄在 18-70 岁之间；Adults aged between 18 to 70 years old;		
2.按 AAO-HNS 诊断标准确诊为特发性突聋伴眩晕主诉者，即***<u>同时符合</u>***以下 A 和 B 者：<br>A. 突发性聋的诊断标准：72h 内突然发生的、原因不明的感音神经性听力损失，在 250、500、1k、2k、4k、8k 中至少相邻的三个频率听力下降≥30dBHL（正常值参考：既往听力学检查结果或对侧耳听力结果）；Diagnosed with unilateral ISSNHL according to the National Institute for Deafness and Communication Disorders (NICDC) criteria: a decrease in hearing of ≥30 decibels (dB), affecting at least 3 consecutive frequencies occurring within a 72-hour period.<br>B. 患者有前庭功能异常主诉（眩晕、不平衡感、一侧偏倒感等）；Reported vertigo/dizziness/imbalance/lateropulsion；		
3.以下至少一项前庭功能检查异常：平衡台，冷热试验，VEMP，甩头试验；Reported abnormal results in at least one of the vestibular function tests (including SOT, caloric test, vHIT, cVEMP and oVEMP);		
4.自觉发病到入组接受治疗时间在 7 天以内；Onset of audio-vestibular symptoms occurred within 7 days;		

5.患者自愿签署知情同意书；Be willing to sign the informed consent of the study		

1.3. 排除标准 Exclusion criteria

以下任一条满足则需排除 Patients who meet *any* of these criteria are *not* eligible for enrollment:

**排除标准 Exclusion criteria**	**是 Yes**	**否 No**
1. 突发性聋在完善检查后存在已知明确的病因，包括听神经瘤、心脑血管意外、外伤、Hunt 综合征等；Definite etiologies are found or highly suspected after clinical evaluations, such as vestibular schwannoma, stroke, trauma or demyelinating disease;		
2. 发病前曾有听力异常及平衡功能障碍者（包括既往突发性耳聋、前庭性偏头痛、梅尼埃病、良性阵发性位置性眩晕、先天性/遗传性/梅毒性听力损失、耳硬化症等）；Diagnosed with a present or previous hearing or balance disorders which might be confused with ISSNHL (history of Meniere’s disease, benign paroxysmal positional vertigo, vestibular neuronitis or vestibular migraine; history of otosclerosis; history of luetic, congenital or genetic hearing loss, etc.)；		
3. 对侧听力异常；Hearing level (evaluated with PTA) in the unaffected ear is abnormal；		
4. 怀疑中枢前庭功能异常；Suspected as central vestibular dysfunction, evaluated by present and previous medical history, physical examination and VNG；		
5. 合并临床不推荐全身系统性激素治疗的疾病的患者（控制不佳的或胰岛素依赖型糖尿病、结核、乙肝或丙肝、胰腺炎、消化道溃疡史、股骨头坏死、慢性肾功能不全等患者）；Present with conditions contraindicated systemic glucocorticoids use, such as tuberculosis, hepatitis C or B infection, active herpes zoster infection or other known human immunodeficiency virus, pancreatitis, insulin-dependent diabetes mellitus, severe osteoporosis, chronic renal insufficiency or gastrointestinal ulcer;		
6. 曾接受高压氧、抗病毒药物等治疗的患者；Having received other systemic etiological treatments for ISSNHL (including hyperbaric oxcygen therapy (HBOT), thrombolytic drugs and antiviral drugs) which may confound the effects of study drugs；		

7. 入组前 3 个月内接受系统性激素治疗时间超过 3 天以上且总剂量超过 180㎎ 强的松者；A history of more than 3 days sufficient systemic glucocorticoids uses (≥1 mg/kg/d) within 3 months which may increase the risk of adverse effects.		
8. 合并其他疾病如中耳炎、严重颈椎疾病、骨折等，无法完成前庭功能各项检查项目的患者；Not appropriate for receiving vestibular function tests due to combination of fracture, inflammatory or suppurative ear disease, severe cervical spondylosis or severe psychotic disorders;		
9. 合并严重器官功能不全的患者；Multiple organ dysfunction or unstable vital signs；		
10. 孕妇及哺乳期妇女；Pregnancy or lactation；		
11. 研究者认为具有其他不适合参加本次临床研究的理由（如预估完成检查难度大、低依从性表现明显等）；Evaluated as unsuitable for the trial for any other reasons by investigators；		

2. 基线 Baseline

2.1. 人口学信息 Demographics

出生日期 Birthdate		年龄 Age	
性别 Gender	男 Male □　女 Female □		
民族 Nationality	汉族 Han □　其他 Others □：______		
职业 Occupation			

2.2. 病史资料 Medical record

**发病时间 Date of the onset of the disease**	_____年___月____日(yyyy-mm-dd)
**发病天数 Days of onset the condition**	______天(days)
**病情描述 Description for the condition**<br>1. 起病 Onset:	

2. 诱因 Predisposition: 无 None □ 感染 Infection□ 外伤 Trauma □

其他 Others □：____________

3. 病变侧 Lesion side: 右耳 Right ear □ 左耳 Left ear □ 双耳 Both ears □

4. 眩晕情况 Vertigo：

1) 眩晕描述 Description of vertigo _____________；(如天旋地转/房子、周围事物不稳/行走倾斜、偏倒感/头重脚轻/头沉重压迫感/眼前发黑等)

2) 眩晕首次发作时间 Time of first onset of vertigo _________；

3) 眩晕与听力下降/耳鸣出现时间先后关系 Vertigo, hearing loss and tinnitus in chronological order _____________；

4) 眩晕持续时间 Duration of vertigo ______________；

5) 是否与体位相关？Position related？是 Yes□ ； 否 No□

6) 其他方面 Others ______________；

5. 合并疾病 Other disease combined：

无 □；自身免疫疾病 Autoimmune disease □；甲状腺相关疾病 Thyroid-related disease □；心脑血管疾病 Cardiovascular and cerebrovascular disease □； 神经系统疾病 Nervous system disease □； 糖尿病 Diabetes □；其他，请说明 Others □ _________

6. 是否伴耳鸣 Tinnitus：有 Yes □ 无 No □；

7. 其他情况 Other condition:

特殊既往史、用药史 Special previous medical history

体格检查 Physical examination

2.3. 听力学检查 Audiology Examinations

检查时间 Time of examination: ______________

纯音测听 Pure Tone Audiometry (PTA)

左 Left	频率/Hz Frequency/Hz	AC/dB	BC/dB	右 Right	频率/Hz Frequency/Hz	AC/dB	BC/dB
	125				125		
	250				250		
	500				500		
	1k				1k		
	2k				2k		
	4k				4k		
	8k				8k		

均值 Average：

左 AC Left AC：_____________ dB；左 BC Left BC：_____________ dB；

右 AC Right AC：_____________ dB；右 BC Right BC：_____________ dB；

声阻抗 Acoustic Impedance

左 Left：A□ B□ C□

右 Right：A□ B□ C□

2.4. 前庭功能检查 Vestibular Function Examinations

1. 视频眼震电图 Videonystagmography (VNG)

检查时间 Time of examination: _____________

温度试验 Caloric Test

1） 双半规管功能：对称 Symmetric □ ；不对称（左侧减弱）Not Symmetric (Left side reduced) □ ；不对称（右侧减弱）Not symmetric (Right side reduced) □

2） 优势偏向 Directional Preponderance：无 None □ ；有（向左侧）Yes (Left side) □ ；有（向右侧）Yes (Right side) □

其他项目异常结果（扫视、追踪、视动、凝视、自发性眼震、变位性眼震、固视抑制）Abnormal results in other items of VNG (Saccade, tracking, visual movement, gaze, nystagmus, fixation suppression):

2. **感觉统合试验 Sensory Organization Test (SOT)**

检查时间 Time of examination: _____________

**本体觉 Proprioceptive sensation**	**视觉 Vision**	**前庭觉 Vestibule**
1. 正常 Normal □<br>2. 不足 Deficiency □<br>A. 前后平衡不足 □<br>B. 侧向平衡不足 □	1. 正常 Normal □<br>2. 不足 Deficiency □<br>A. 前后平衡不足 □<br>B. 侧向平衡不足 □	1. 正常 Normal □<br>2. 不足 Deficiency □<br>A. 前后平衡不足 □<br>B. 侧向平衡不足 □

总体平衡 Overall balance:

1.良好 Excellence □ 2.稍差 A little bit worse□

3.较差 Rather worse □ 4.易跌倒 Easy to fall down

3. 头脉冲试验 Video Head Impulse Test (vHIT)

检查时间 Time of examination: _____________

**部位 Site**	**增益 Gain**	**病理性扫视波 Abnormal wave**
左水平半规管<br>Left horizontal semicircular canal		有 Yes □；无 No □；
右水平半规管<br>Right horizontal semicircular canal		有 Yes □；无 No □；
左前半规管<br>Left anterior semicircular canal		有 Yes □；无 No □；
右后半规管<br>Right posterior semicircular canal		有 Yes □；无 No □；

左后半规管<br>Left posterior semicircular canal		有 Yes □；无 No □；
右前半规管<br>Right anterior semicircular canal		有 Yes □；无 No □；

4. 前庭诱发肌源性电位 Vestibular Evoked Myogenic Potentials (VEMP)

检查时间 Time of examination: ______________

1) 眼前庭诱发肌源性电位(oVEMP) Ocular vestibular evoked myogenic potentials

左侧 Left side： 1.未引出 No response □ 2.引出 Response □

引出阈值 Response threshold：___________dB nHL

右侧 Right side： 1.未引出 No response □ 2.引出 Response □

引出阈值 Response threshold：___________dB nHL

2) 颈肌前庭诱发肌源性电位(cVEMP) Cervical Vestibular evoked myogenic potentials

左侧 Left side： 1.未引出 No response □ 2.引出 Response □

引出阈值 Response threshold：___________dB nHL

右侧 Right side： 1.未引出 No response □ 2.引出 Response □

引出阈值 Response threshold：___________dB nHL

2.5. 影像学检查 Imaging Examination

MRI

2.6. 眩晕障碍量表 Dizziness Handicap Inventory (DHI)

患者编号	生理部分得分	情绪部分得分	功能部分得分

填写日期 Date：

2.7. 视觉模拟评分 Visual Analog Scale（VAS）

评分（0-10）：

填写日期 Date：

----

3. 随访一：治疗一周后随访

3.1. 听力学检查 Audiology Examinations

检查时间 Time of examination: ____________

纯音测听 Pure Tone Audiometry (PTA)

左 Left	频率/Hz Frequency/Hz	AC/dB	BC/dB	右 Right	频率/Hz Frequency/Hz	AC/dB	BC/dB
	125				125		
	250				250		
	500				500		
	1k				1k		
	2k				2k		
	4k				4k		
	8k				8k		

均值：

左 AC Left AC：____________ dB；左 BC Left BC：____________ dB；

右 AC Right AC：____________ dB；右 BC Right BC：____________ dB；

3.2. 眩晕障碍量表 Dizziness Handicap Inventory (DHI)

患者编号	生理部分得分	情绪部分得分	功能部分得分

填写日期 Date:

3.3. 视觉模拟评分 Visual Analog Scale（VAS）

评分（0-10）：

填写日期 Date:

---

4. 随访二 ：治疗开始 2 周后随访

4.1. 听力学检查 Audiology Examinations

检查时间 Time of examination: ____________

纯音测听 Pure Tone Audiometry (PTA)

左 Left	频率/Hz Frequency/Hz	AC/dB	BC/dB	右 Right	频率/Hz Frequency/Hz	AC/dB	BC/dB
	125				125		
	250				250		
	500				500		
	1k				1k		
	2k				2k		
	4k				4k		
	8k				8k		

均值/dB HL：

左 AC Left AC：____________ dB；左 BC Left BC：____________ dB；

右 AC Right AC：____________ dB；右 BC Right BC：____________ dB；

4.2. 眩晕障碍量表 Dizziness Handicap Inventory (DHI)

患者编号	生理部分得分	情绪部分得分	功能部分得分

填写日期 Date:

4.3. 视觉模拟评分 Visual Analog Scale（VAS）

评分（0-10）：

填写日期 Date：

---

5. 随访三 ：治疗开始 4 周后随访

5.1. 听力学检查 Audiology Examinations

检查时间 Time of examination: ______________

纯音测听 Pure Tone Audiometry (PTA)

左 Left	频率/Hz Frequency/Hz	AC/dB	BC/dB	右 Right	频率/Hz Frequency/Hz	AC/dB	BC/dB
	125				125		
	250				250		
	500				500		
	1k				1k		
	2k				2k		
	4k				4k		
	8k				8k		

均值/dB HL：

左 AC Left AC：______________ dB；左 BC Left BC：______________ dB；

右 AC Right AC：______________ dB；右 BC Right BC：______________ dB；

5.2. 前庭功能检查 Vestibular Function Examinations

1. 视频眼震电图 Videonystagmography (VNG)

检查时间 Time of examination: ______________

温度试验 Caloric Test

1） 双半规管功能：对称 Symmetric ☐ ；不对称（左侧减弱）Not Symmetric (Left side reduced) ☐ ；不对称（右侧减弱）Not symmetric (Right side reduced) ☐

2） 优势偏向 Directional Preponderance：无 None ☐ ；有（向左侧）Yes (Left side) ☐ ；有（向右侧）Yes (Right side) ☐

其他项目异常结果（扫视、追踪、视动、凝视、自发性眼震、变位性眼震、固视抑制）Abnormal results in other items of VNG (Saccade, tracking, visual movement, gaze, nystagmus, fixation suppression):

2. **感觉统合试验 Sensory Organization Test (SOT)**

检查时间 Time of examination: ______________

**本体觉 Proprioceptive sensation**	**视觉 Vision**	**前庭觉 Vestibule**
3. 正常 Normal ☐<br>4. 不足 Deficiency ☐<br>C. 前后平衡不足 ☐<br>D. 侧向平衡不足 ☐	3. 正常 Normal ☐<br>4. 不足 Deficiency ☐<br>C. 前后平衡不足 ☐<br>D. 侧向平衡不足 ☐	3. 正常 Normal ☐<br>4. 不足 Deficiency ☐<br>C. 前后平衡不足 ☐<br>D. 侧向平衡不足 ☐

总体平衡 Overall balance:

1.良好 Excellence ☐ 2.稍差 A little bit worse☐

3.较差 Rather worse ☐ 4.易跌倒 Easy to fall down ☐

3. 头脉冲试验 Video Head Impulse Test (vHIT)

检查时间 Time of examination: ______________

**部位 Site**	**增益 Gain**	**病理性扫视波 Abnormal wave**
左水平半规管<br>Left horizontal semicircular canal		有 Yes ☐；无 No ☐；
右水平半规管<br>Right horizontal semicircular canal		有 Yes ☐；无 No ☐；
左前半规管		有 Yes ☐；无 No ☐；

Left anterior semicircular canal		
右后半规管<br>Right posterior semicircular canal		有 Yes □；无 No □；
左后半规管<br>Left posterior semicircular canal		有 Yes □；无 No □；
右前半规管<br>Right anterior semicircular canal		有 Yes □；无 No □；

4. 前庭诱发肌源性电位 Vestibular Evoked Myogenic Potentials (VEMP)

检查时间 Time of examination: ______________

1) 眼前庭诱发肌源性电位(oVEMP) Ocular vestibular evoked myogenic potentials

左侧 Left side： 1.未引出 No response □ 2.引出 Response □

引出阈值 Response threshold：__________dB nHL

右侧 Right side： 1.未引出 No response □ 2.引出 Response □

引出阈值 Response threshold：__________dB nHL

2) 颈肌前庭诱发肌源性电位(cVEMP) Cervical Vestibular evoked myogenic potentials

左侧 Left side： 1.未引出 No response □ 2.引出 Response □

引出阈值 Response threshold：__________dB nHL

右侧 Right side： 1.未引出 No response □ 2.引出 Response □

引出阈值 Response threshold：__________dB nHL

5.3. **眩晕障碍量表** Dizziness Handicap Inventory (DHI)

患者编号	生理部分得分	情绪部分得分	功能部分得分

填写日期 Date：

5.4. 视觉模拟评分 Visual Analog Scale（VAS）

评分（0-10）：

填写日期 Date：

---

6. 随访四 ：治疗开始 8 周后随访

6.1. 听力学检查 Audiology Examinations

检查时间 Time of examination: ______________

纯音测听 Pure Tone Audiometry (PTA)

左 Left	频率/Hz Frequency/Hz	AC/dB	BC/dB	右 Right	频率/Hz Frequency/Hz	AC/dB	BC/dB
	125				125		
	250				250		
	500				500		
	1k				1k		
	2k				2k		
	4k				4k		
	8k				8k		

均值/dB HL：

左 AC Left AC：______________ dB；左 BC Left BC：______________ dB；

右 AC Right AC：______________ dB；右 BC Right BC：______________ dB；

6.2. 前庭功能检查 Vestibular Function Examinations

1. 视频眼震电图 Videonystagmography (VNG)

检查时间 Time of examination: ______________

温度试验 Caloric Test

1） 双半规管功能：对称 Symmetric □ ；不对称（左侧减弱）Not Symmetric (Left side reduced) □ ；不对称（右侧减弱）Not symmetric (Right side reduced) □

2） 优势偏向 Directional Preponderance：无 None □ ；有（向左侧）Yes (Left side) □ ；有（向右侧）Yes (Right side) □

其他项目异常结果（扫视、追踪、视动、凝视、自发性眼震、变位性眼震、固视抑制）Abnormal results in other items of VNG (Saccade, tracking, visual movement, gaze, nystagmus, fixation suppression):

2. **感觉统合试验 Sensory Organization Test (SOT)**

检查时间 Time of examination: ______________

**本体觉 Proprioceptive sensation**	**视觉 Vision**	**前庭觉 Vestibule**
5. 正常 Normal □<br>6. 不足 Deficiency □<br>E. 前后平衡不足 □<br>F. 侧向平衡不足 □	5. 正常 Normal □<br>6. 不足 Deficiency □<br>E. 前后平衡不足 □<br>F. 侧向平衡不足 □	5. 正常 Normal □<br>6. 不足 Deficiency □<br>E. 前后平衡不足 □<br>F. 侧向平衡不足 □

总体平衡 Overall balance:

1.良好 Excellence □　2.稍差 A little bit worse□

3.较差 Rather worse □　4.易跌倒 Easy to fall down □

3. 头脉冲试验 Video Head Impulse Test (vHIT)

检查时间 Time of examination: ______________

**部位 Site**	**增益 Gain**	**病理性扫视波 Abnormal wave**
左水平半规管<br>Left horizontal semicircular canal		有 Yes □；无 No □；
右水平半规管<br>Right horizontal semicircular canal		有 Yes □；无 No □；
左前半规管		有 Yes □；无 No □；

Left anterior semicircular canal		
右后半规管<br>Right posterior semicircular canal		有 Yes □；无 No □；
左后半规管<br>Left posterior semicircular canal		有 Yes □；无 No □；
右前半规管<br>Right anterior semicircular canal		有 Yes □；无 No □；

4. 前庭诱发肌源性电位 Vestibular Evoked Myogenic Potentials (VEMP)

检查时间 Time of examination: ______________

1) 眼前庭诱发肌源性电位(oVEMP) Ocular vestibular evoked myogenic potentials

左侧 Left side： 1.未引出 No response □ 2.引出 Response □

引出阈值 Response threshold：__________dB nHL

右侧 Right side： 1.未引出 No response □ 2.引出 Response □

引出阈值 Response threshold：__________dB nHL

2) 颈肌前庭诱发肌源性电位(cVEMP) Cervical Vestibular evoked myogenic potentials

左侧 Left side： 1.未引出 No response □ 2.引出 Response □

引出阈值 Response threshold：__________dB nHL

右侧 Right side： 1.未引出 No response □ 2.引出 Response □

引出阈值 Response threshold：__________dB nHL

6.3. 眩晕障碍量表 Dizziness Handicap Inventory (DHI)

患者编号	生理部分得分	情绪部分得分	功能部分得分

填写日期 Date：

6.4. 视觉模拟评分 Visual Analog Scale（VAS）

评分（0-10）：

填写日期 Date：

---

7. 研究结束

7.1. 研究总结

1. **受试者是否完成临床试验期？如为“否”，请填写中止试验原因 Has the participant completed the study per the protocol?**

   是 Yes □　否 No □

2. **如是，**

   1) **中止日期 Date of suspended the study ____________________;**

   2) **试验期中止治疗的主要原因（选择一个） The reason of the suspending:**

被主研单位中止 Suspended by the organizer □;

出现不良事件 Adverse events □___________;

缺乏疗效 No efficacy □;

虽然未出现不良事件，但出于安全性考虑，研究者认为终止治疗对受试者最有益 The investigator made decision □;

患者撤回知情同意书 The participant cancealed the informed concent □;

失访（包括患者自行退出）Loss of follow-up □;

妊娠 Pregnancy □;

死亡 Death □;

其它原因(请描述）Other reason □ ________

---

8. 声明

8.1. 监察员声明 Monitor statement

经监查，本病例报告表的各项内容均符合《药物临床试验质量管理规范》（GCP）和研究方案的要求，所有信息都是真实和完整的。

监查员签名 Signature of the monitor

日期 Date of signature

8.2. 课题负责人声明 PI's statement

我已经审阅过此病例报告表中的全部内容和数据，经核实，确认信息记录真实、准确、科学、规范，项目填写完整，符合试验方案的要求。

研究员签名 Signature of the researcher

日期 Date of signature

表格完成人 **Form Completed By: ____________________________**

完成时间 **Date: ________**

附件：

DHI、VAS 量表问卷（微信扫描二维码）V2.0

DHI&VAS Wechat Code V2.0

知情同意书

(Consent Form)

尊敬的先生/女士：

我们将邀请您参加一项临床研究。题目是“**激素治疗特发性突聋伴眩晕患者的前庭功能转归研究**”。在您参加此项研究之前，请您仔细阅读这份知情同意书并慎重做出是否参加本项研究的决定。您可以向您的研究医生/研究人员询问任何您不懂的地方，让他/她给您解释，直到您完全理解为止。您在做出参加此项研究的决定之前，可以和您的家人及朋友进行充分的讨论。若您正在参加别的研究，请告知您的研究医生或研究人员，本研究的主要内容如下：

一、研究背景：

1、相关研究进展：特发性突发性感音神经性聋（简称特发性突聋）是目前突发性聋中最常见的类型（84%-89%），发病率约在 5-20/100,000 人每年，其中约 28%-57%的患者被报道伴有不同程度的眩晕表现，多数研究认为伴眩晕表现提示 ISSNHL 患者不良预后。近年来随着前庭功能检测手段不断发展进步，前庭损伤的具体定位变得越来越准确。激素治疗是目前治疗特发性突聋的标准方案，包括口服激素系统性给药以及鼓室内注射激素局部给药。但不同激素治疗后患者前庭功能恢复与转归情况目前尚缺乏高质量临床观察证据。

2、课题性质：本研究为复旦大学附属眼耳鼻喉科医院耳鼻喉科研究院组织实施，课题起止时间是 2019 年 9 月 至 2020 年 12 月。

3、研究目的：研究评估突发性耳聋伴眩晕患者的前庭功能，探索眩晕发生、前庭代偿和适应的相关机制，研究不同激素治疗在促进前庭康复中的效果。

4、此项研究已经得到复旦大学附属眼耳鼻喉科医院伦理委员会的审批，将遵从赫尔辛基宣言等保护受试者权益的国际原则和中国国家相关法律、法规，符合医学伦理道德。

二、研究设计和研究过程：

1、这项研究将针对性收集特发性突发性耳聋伴眩晕表现的患者资料，对他们治疗前、治疗中及治疗后前庭功能、听力损失方面客观检查数据，以及眩晕、耳鸣主观症状量表进行比较和统计分析。

2、如果您自愿参加此项研究，你将被随机分配到口服激素（强的松）或鼓室内注射激素（甲强龙）两综合治疗组中的任意一组。我们将需要您按照研究方案的要求在规定时间内（治疗开始后第 1 周、第 2 周、第 4 周、第 8 周）来我院完成上述主观的评估量表及客观的评估检查。这些量表和检查都是无创性的评估方法，也不会影响您正常的诊断和治疗。

3、当我们收集好这些资料后，我们将建立专门的资料库，入组患者使用项目统一编号，个人资料和病例信息由样本管理的专人录入、保存，使用者看到的仅为个体编号，不再接触参加者的姓名等资料。并由专人对这些结果进行比较和统计学分析。

4、个人隐私的保密措施：您的医疗记录将保存在医院，仅供研究人员查阅。必要时，政府管理部门或伦理委员会的成员按规定可以查阅您的个人资料。研究结果将以经统计分析后的数据形式发表，不包含任何可识别的受试者信息。

三、可能的风险和受益：

1、**可能的风险：**

本项研究为干预性研究，干预方式均依据目前国内外指南标准建议激素治疗剂量和方案进行，分别为口服激素治疗与鼓室内注射激素治疗。激素药物均为已上市治疗药物。

1)　　、激素药物的副作用（包括体重增加等代谢异常、感染、骨质疏松、加

重原有消化道溃疡、鼓室注射相关鼓膜穿孔等）。您的研究人员将会监控激素药物的副作用，若试验期间您发生任何副作用或不适，请立刻向研究人员报告，研究人员会对您反映的情况作出医学评估，如果您或您的研究人员认为您无法耐受这些副作用，研究药物可能会完全停用。

2）　、如果您因参与这项研究而受到伤害，且该损害是不参加本研究而接受正常治疗所不会发生的，您可以获得免费治疗，以及必要时将根据中国法律获得相应的补偿。

3）　、您在研究期间需要按时到医院随访，这将占用您的一些时间，给您造成麻烦或带来不便敬请谅解；交通不便的患者，我们会适当予以交通补助或安排住院接受治疗。

2、**可能的受益：**

1）　、定期随访将有利于您的病情监测，以及听力、前庭损伤情况的具体分析，为您疾病预后的转归提供更多临床证据帮助。

2）　、通过对您的样本进行检测将有助于未来明确突发性耳聋伴眩晕的病因或病变部位并进行有效的治疗，减少该疾病的发生率、降低严重程度或明显缩短病程。

3）　、参与该研究可以帮助减免您的部分检查、治疗费用，但研究的结果并不一定能够直接用于您的诊断和治疗。

4）　、我们会在一定范围内优先为您安排检查，但需要在综合考虑其他患者权益为前提下进行。

3、**作为研究受试者，您有以下职责**：提供有关自身病史和当前身体状况的真实情况；告诉研究医生自己在本次研究期间所出现的任何不适；不得私自服用受

限制的药物、食物等；告诉研究医生自己在最近是否曾参与其他研究，或目前正参与其他研究；定期完成随访；遵循研究人员的指导。

四、自愿参加：

您参与研究是完全自愿的。您可以选择不参加本项研究，或者在任何时候通知研究者要求退出研究，您的数据将不纳入研究结果，您的任何医疗待遇与权益不会因此而受到影响。当您决定不再参加本研究时，希望您及时告知您的研究人员，研究人员可就您的健康状况提供建议和指导。

申办方或者监管机构也可能在研究期间终止本研究。如果发生本研究提前终止的情况，我们将及时通知您，您的研究人员会根据您的健康状况为您下一步的治疗计划提供建议。

如果不参加本研究，您的其余治疗方法选择有：静脉使用激素治疗、药物改善血液循环及保护神经（金纳多、甲钴胺）、高压氧治疗等。

五、研究费用：参加此项研究不会增加您的任何费用，所有前庭功能相关检查费用及治疗费用均由研究者负责。如您于外地居住，我们可根据具体情况考虑为您提供住院，或者给予一定交通补助。如果您选择中途退出研究或者私自接受研究以外相关治疗，则视为退组或治疗方案变异，需要向研究组补交除前庭检查费用以外的全体检查、治疗费用，并且研究组将不再负责您后续的治疗检查费用，请您明确。

六、联系人及联系方式：如果您本人对此项研究有任何疑问，可直接与复旦大学附属眼耳鼻喉科医院郝维明医生联系。

联系电话：

七、该知情同意书一式两份各 4 页，双方妥善保管，在双方签字后有效。

知情同意书签字页

参试者知情同意声明

我已阅读上述信息并理解该研究的目的以及参加该研究所带来的潜在益处，我对研究程序、研究内容提出的所有问题均已得到令我满意的答复。我也被告知，当我有问题或想进一步获得信息，应当与谁联系。我自愿签署这份知情同意书，并自愿参加这项研究。

参试者签字：　　　　　　　　　　　　　　　　**签字日期：**

参试者联系电话：

研究人员告知声明

我们已经向研究对象宣读和解释了这份知情同意书，并且回答了他/她所提出的所有问题。

他/她本人也已经理解并同意参加此项临床科学研究。

研究者签字：　　　　　　　　　　　　　　　　**签字日期：**

研究人员联系电话：
