# Supplementary material for: Vestibular prognosis in idiopathic sudden sensorineural hearing loss with vestibular dysfunction treated with oral or intratympanic glucocorticoids: a protocol for randomized controlled trial
Source: Trials. 2020 Jul 22;21:669. doi: 10.1186/s13063-020-04579-6 (PMC7477872; doi:10.1186/s13063-020-04579-6)
Supplement: Supplementary file 3 — Additional file 3. Adverse Event Forms Template. [file 13063_2020_4579_MOESM3_ESM.pdf]

不良事件监测 ADVERSE EVENTS

中心编号:　　患者编号:　　填写日期:

**不良事件名称 Adverse event name**	
**严重程度 Intensity**	☐ 轻度 Mild ☐ 中度 Moderate ☐ 重度 Severe
**如发生严重不良事件请说明 If SAE specify:**	☐ 死亡 Death<br>☐ 威胁生命的 Life-threatening<br>☐ 持续或有症状的残疾或无行为能力 Persistent or symptomatic disability or incapacity<br>☐ 因不良事件住院或延长治疗时间 Hospitalisation or prolongation of treatment<br>☐ 其他重要医疗事件 Other important medical event ________
**发生时间 Onset Date**	（YYYY-MM-DD）
**结束时间 End Date**	**或 OR** ☐ 持续到研究结束 Ongoing at the end of study
**治疗方式 Therapy**	☐ 无 None ☐ 药物，请说明药物名称 Drug ________________<br>☐ 其他，请说明 Other _ ☐ 药物及其他，请说明 Drug and other __________
**研究干预调整 Action Taken with Interventions**	☐ 干预方式无改变 Intervention unchanged ☐ 干预频次减少 Frequency reduced<br>☐ 暂时中止治疗 Intervention temporarily interrupted ☐ 停止干预 Intervention withdrawn<br>☐ 未知 Not Known<br>☐ 干预频次增加 Frequency increased
**研究用药调整 Action Taken with Study Drug**	☐ 剂量无改变 Dose unchanged ☐ 剂量减少 Dose reduced<br>☐ 暂时中止用药 Drug temporarily interrupted ☐ 停止用药 Drug withdrawn<br>☐ 剂量增加 Dose increased ☐ 未知 Not Known
**结局 Outcome**	☐ 完全恢复 Recovered ☐ 正在恢复中 Recovering<br>☐ 恢复，但留有后遗症 Recovering with sequelae ☐ 症状仍然存在 Continuing<br>☐ 致死 Fatal ☐ 未知 Not Known
**不良事件与研究干预相关性 Relationship to Study drug/ interventions**	☐ 确定 Certain ☐ 很可能 Probable ☐ 可能 Possible<br>☐ 不太可能 Unlikely ☐ 不相关 Not related ☐ 无法确认 Unclassified
